# Missed opportunities for guidance on sexually transmitted infection services: a global review of national HIV PrEP guidance

**DOI:** 10.1136/sextrans-2023-056081

**Published:** 2024-06-19

**Authors:** Erica Spielman, Maeve B Mello, Robin Schaefer, Jason Ong, Heather-Marie A Schmidt, Mary Henderson, Pietro Vinti, Mateo Prochazka, Niklas Luhmann, Rachel Baggaley

**Affiliations:** 1 Global HIV, Hepatitis, and STIs Programmes, World Health Organization, Geneva, Switzerland; 2 Forum for Collaborative Research, Berkeley, California, USA; 3 Central Clinical School, Monash University, Melbourne, Victoria, Australia; 4 Independent consultant, Riverside, Connecticut, USA; 5 Joint Infectious Diseases Unit, World Health Organization Regional Office for Europe, Copenhagen, Capital Region, Denmark

**Keywords:** Pre-Exposure Prophylaxis, REPRODUCTIVE HEALTH, SEXUAL HEALTH

## Abstract

**Objectives:**

People who use or would benefit from pre-exposure prophylaxis (PrEP) for HIV infection are disproportionately affected by sexually transmitted infections (STIs). Integrating STI services when offering PrEP fosters synergies and efficiencies in response to HIV/STI and promotes people-centred care. Including guidance on STI interventions for people on PrEP may facilitate implementation and uptake. We conducted a global review of national PrEP guidance documents and analysed the inclusion of recommendations for the provision of STI services by country level of income.

**Methods:**

We searched national PrEP guidance documents published by WHO Member States through the WHO, the Joint United Nations Programme on HIV/AIDS (UNAIDS) databases, the *PrEPWatch* repository and Google. Information on a range of STI-related interventions was extracted from documents available by October 2023.

**Results:**

Of the 113 national PrEP guidance documents retrieved, STIs were mentioned in 77% (90/117). Viral hepatitis B testing and vaccination were recommended by most high-income countries (HICs) and low-income and middle-income countries (LMICs). Recommendation for syphilis testing was prominent in HICs (91%) and moderately noted in LMICs (68%). Gonorrhoea and chlamydia testing was recommended frequently in HICs (88%) and 42% in LMICs. However, the review noted that, to a much lesser extent, specific type of testing for these pathogens was mentioned. Recommendation for quarterly STI testing for syphilis, gonorrhoea and chlamydia was ubiquitous, while the need to offer STI partner services was rarely mentioned.

**Conclusions:**

PrEP services offer an opportunity for improved and expanded STI services, increasing person-centred care and addressing STI epidemics alongside HIV. Our review highlights the strengths and gaps in incorporating critical STI interventions into national PrEP normative guidance. Addressing these gaps through a stepwise approach and increasing targeted testing and partner services can help improve quality of care and support an effective response to HIV and other STIs.

**WHAT IS ALREADY KNOWN ON THIS TOPIC**

- Globally, little progress has been made in curbing the epidemics of sexually transmitted infections (STIs).
- STI prevalence among people initiating pre-exposure prophylaxis (PrEP) for HIV infection and STI incidence while taking PrEP has been demonstrated to be high and much higher than the estimates for the general population.
- Integrating STI services when offering PrEP for those disproportionately affected may foster synergies and efficiencies in the response to HIV and STI epidemics and promote people-centred care.

**WHAT THIS STUDY ADDS**

- To the best of our knowledge, this is the first global review of national STI services recommendations for people seeking or taking PrEP.
- The results of this review quantify and highlight the gaps and missed opportunities in national guidance documents to address, prioritise and integrate recommendations related to STI testing, treatment and partner services in the context of PrEP.
- Furthermore, this study provides baseline data to monitor the existence and levels of integration of STI and PrEP services globally and by country income level.

**HOW THIS STUDY MIGHT AFFECT RESEARCH, PRACTICE OR POLICY**

- The findings are expected to (1) raise awareness of policy makers, donors, programme managers, providers and the community regarding the need to address STIs for those at higher risk of infection; (2) prompt action by national HIV programmes to address STIs when PrEP services are recommended; and (3) highlight WHO-recommended STI interventions that could be integrated into PrEP services within a person-centred approach.
- Focusing on people at higher risk of STIs, including HIV, should support reaching the global targets to end STIs as a public health problem by 2030, and improve the quality of life and care of those at substantial risk of HIV/STI acquisition and transmission.

## Introduction

Except for congenital syphilis, until 2020 there has been no progress in the global sexually transmitted infection (STI) response, and many infections remain undiagnosed and untreated.^1^ Most of the 2020 global targets were missed and are currently off-track for those set to 2030.^1 2^ In 2020, the WHO estimated 374 million new infections occurring with one of four curable STIs—syphilis, chlamydia, gonorrhoea and trichomoniasis—among those aged 15–49 years.^1^ Many STIs can be asymptomatic or unrecognised by both individuals and health providers; if left untreated, some can have serious sequelae or death.^1^

Since 2015, the WHO has recommended multiple HIV pre-exposure prophylaxis (PrEP) options to people at substantial risk of infection as part of combination HIV prevention in the context of broader sexual health services, including STIs. PrEP services are a key opportunity for offering STI testing and treatment, as people who could benefit from prophylaxis for HIV infection are also at increased risk of other STIs.^3 4^ Although overall the incidence and prevalence of STIs is high in people requesting and taking PrEP, there is significant heterogeneity among PrEP clients.^5^ A global systematic review and meta-analysis found that 24% of individuals initiating PrEP were diagnosed with any of three curable STIs (ie, syphilis, chlamydia and/or gonorrhoea) and pooled STI incidence remained high during PrEP follow-up visits.^6^ Similarly, high STI baseline prevalence was found among young African women (age 18–25) from three PrEP cohorts, with 29% of them diagnosed with chlamydia and 11% for gonorrhoea.^7^

Modelling studies support the proposition that integrating STI services into PrEP can have a significant impact on STI incidence.^8^ Following a slow start, as of 2021, 144 countries reported adaptation of the WHO recommendations on oral PrEP in national guidelines and an additional 14 countries reported to the UNAIDS/WHO Global AIDS Monitoring (GAM) platform that they plan to adopt the recommendations in the next 2 years. Approximately 1.6 million people globally received oral PrEP at least once during 2021.^9^ The rapid increase in the number of people using PrEP highlights the need to actively integrate STI services within these programmes to treat and prevent STIs.

Given the progress in PrEP implementation and scale-up, there is an opportunity to highlight and strengthen integration of STI services for those at higher risk of both HIV and other STIs towards more person-centred care. To support such integration, we conducted a review of national PrEP guidance documents and analysed the inclusion of recommendations of STI services for people seeking or taking PrEP.

## Methods

A database of existing national PrEP guidance documents from among the 194 WHO Member States (referred to here as countries) was collated from the WHO Global HIV, Hepatitis and STIs Programmes database and from the web portal *PrEPWatch*.^10^ Additionally, a Google search was conducted for missing documents using name of country + ‘HIV guidelines’ or ‘PrEP’ or ‘pre-exposure prophylaxis’ in the official national language. Finally, staff from WHO Regional Offices were contacted to assist in providing additional national PrEP guidance documents from countries where no information was retrieved. The latest version of each guidance document was used for data extraction.

A variety of PrEP guidance document types were included in this review such as national stand-alone PrEP guidance documents, protocols, frameworks, implementation guidelines, HIV national strategic plans, HIV or antiretroviral guidelines, testing and/or clinical guidelines and standard operating procedures.

Documents written in languages other than English were translated using ‘Google translate’. For countries with stand-alone PrEP guidance, the document was reviewed in full. Where PrEP was included as part of broader HIV guidelines, relevant key words were used to detect if any STI-related intervention was mentioned. For national documents for which Google translation was incomprehensible to the authors, the key words were back translated, and searches performed in the original language of the document. If search words were detected, the identified section containing PrEP guidance was translated.

Guidance documents were included for data extraction if they (1) were from a WHO Member State, (2) provided guidance for PrEP implementation and (3) mentioned STIs in the context of PrEP services. Information on type of STI case management, tests used for each pathogen, treatment recommendations, vaccinations, among other themes, was extracted following the variables described in online supplemental file 1. We did not collect information on hepatitis C screening in the data collection. Data extraction was conducted by two independent researchers with a reviewer to resolve any discrepancies. When relevant, narratives were extracted to provide content for data interpretation.

10.1136/sextrans-2023-056081.supp1 Supplementary data

Data were analysed and presented based on frequency distribution of relevant variables globally and by country level of income based on the World Bank classification.

### Patient and public involvement

No patients were involved in this study. Data were solely collected from publicly available guidance documents.

## Results

**Figure 1:**
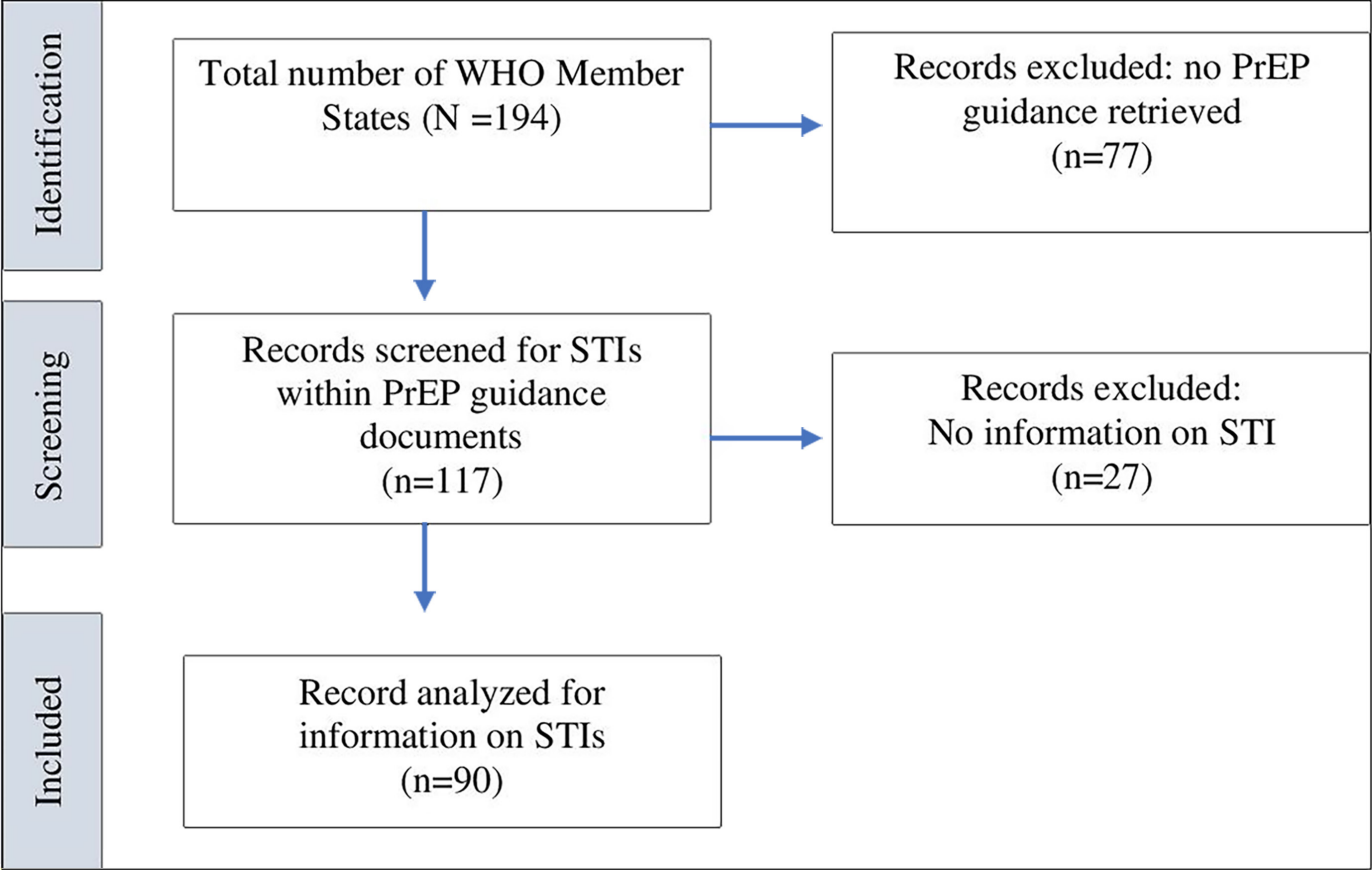
PRISMA (Preferred Reporting Items for Systematic Reviews and Meta-Analyses) flow diagram. PrEP, pre-exposure prophylaxis; STI, sexually transmitted infection.

Out of the 194 WHO Member States, 117 (60%) national PrEP guidance documents were retrieved. Of these, 27 were excluded as STIs were not mentioned. The remaining 90 (77%) were reviewed in full (figure 1).

At the time of this review, from the retrieved documents, almost all countries in the South-East Asia region had guidance for PrEP implementation but only 60% of those documents mentioned STIs. In the Americas and Western Pacific regions, a lower proportion of countries have PrEP guidelines, 51% and 41%, respectively and, when where they exist, STIs were mentioned in almost all of them (table 1).

**Table 1:** Number of countries with national HIV pre-exposure prophylaxis (PrEP) documents retrieved and number of retrieved national guidance documents which mention sexually transmitted infection (STI) services, by WHO regions (October 2023)

WHO region	Total number of countries in each region	Countries with PrEP guidance documents retrieved n (%)	Countries with PrEP guidance documents retrieved which mentioned STIs n (%)
South-East Asia	11	10 (91)	6 (60)
Europe	53	43 (81)	34 (79)
Africa	47	32 (68)	22 (69)
Americas	35	18 (51)	16 (89)
Western Pacific	27	11 (41)	10 (91)
Eastern Mediterranean	21	3 (14)	2 (67)
**Total**	**194**	**117 (60)**	**90 (77)**

Packages of services for STIs within PrEP services varied among countries. Some guidance documents only mentioned ‘screen for STIs’ with limited information of types of STI case management, pathogen or tests, while others presented detailed information. Of the total WHO member states where PrEP guidance documents included STIs services, 57 (41%) out of 139 were from low-income and middle-income countries (LMICs) and 33 (60%) out of 55 from high-income countries (HICs). Almost half of them (40/90) referred users to the STI national guidelines to complement the information provided in the PrEP guidance documents. Only one (India) included detailed guidance on STI case management in the PrEP guidance document. Tables 2–3 summarise the types of STI interventions mentioned in the documents analysed by country income level.

**Table 2:** Overview of STI services in national PrEP guidance documents retrieved, by country income level

Overview of STI services	HIC (%) n=33	LMIC (%) n=57
**Type of STI case management**		
**Any type of STI case management**	**24/33 (73)**	**45/57 (79)**
Syndromic only	0/33 (0)	7/57 (12)
Syndromic plus syphilis testing	0/33 (0)	14/57 (25)
Aetiologic only	20/33 (61)	8/57 (14)
Both (syndromic+aetiologic when available)	4/33 (12)	16/57 (28)
Type not specified	9/33 (27)	12/57 (21)
**Recommended STI screening**		
HBV	31/33 (94)	55/57 (96)
Syphilis	30/33 (91)	39/57 (68)
NG/CT	29/33 (88)	24/57 (42)
**Recommended vaccinations**		
HBV	29/33 (88)	36/57 (63)
HPV	10/33 (30)	8/57 (14)
**Recommended STI partner services**	**9/33 (27)**	**5/57 (9)**
**Recommended NG AMR testing**	**0 (0)**	**0 (0)**

AMR, antimicrobial resistance; CT, *Chlamydia trachomatis*; HBV, hepatitis B virus; HIC, high-income country; HPV, human papillomavirus; LMIC, low-income and middle-income country; NG, *Neisseria gonorrhoeae*; PrEP, pre-exposure prophylaxis; STI, sexually transmitted infection.

**Table 3:** Syphilis, gonorrhoea and chlamydia testing in national PrEP guidance documents retrieved, by country income level

Syphilis screening	HIC (%) n=30	LMIC (%) n=39
Recommended lab-based test type		
Serological tests (non-specified)	14/30 (47)	5/39 (13)
Treponemal plus non-treponemal	4/30 (13)	13/39 (33)
Non-treponemal test only	2/30 (7)	7/39 (18)
Treponemal test only	0/30 (0)	2/39 (5)
Type not specified	10/30 (33)	12/39 (31)
Recommended rapid test type		
Single treponemal	0/30 (0)	10/39 (26)
Dual HIV/syphilis (treponemal)	0/30 (0)	2/39 (5)
Recommended screening frequency		
Every 3 months	19/30 (63)	21/39 (54)
Every 3–6 months	5/30 (17)	3/39 (8)
Every 6 months	5/30 (17)	7/39 (18)
Other	1/30 (3)	3/39 (8)
Frequency not specified	0/30 (0)	5/39 (13)
**NG/CT screening**	**HIC (%)** **n=29**	**LMIC (%)** **n=24**
Molecular testing recommended	19/29 (66)	9/24 (38)
Sampling recommended for all relevant anatomical sites*	18/29 (62)	8/24 (33)
Recommended screening frequency		
Every 3 months	17/29 (59)	13/24 (54)
Every 3–6 months	6/29 (21)	3/24 (13)
Every 6 months	4/29 (14)	6/24 (25)
Other	0/29 (0)	1/24 (4)
Frequency not specified	1/29 (3)	1/24 (4)

*Urethra, vaginal/cervix, anorectum and oropharynx, as applicable.

CT, *Chlamydia trachomatis*; HIC, high-income country; LMIC, low-income and middle-income country; NG, *Neisseria gonorrhoeae*; PrEP, pre-exposure prophylaxis.

### Types of STI case management

In LMICs, 79% (45/57) of the national guidance documents retrieved specified the type of STI management. Twelve per cent (7/57) recommended syndromic case management alone, 25% (14/57) recommended syndromic approach but with the addition of syphilis testing, 14% (8/57) mentioned aetiological management and 28% (16/57) recommended a combination of syndromic and aetiological case management without specifying types of pathogens. Conversely, no national guidance documents from HICs recommended syndromic case management alone.

### STI testing

Hepatitis B (HBV) testing was recommended by many national guidance documents from both HICs and LMICs, 94% (31/33) and 96% (55/57), respectively.

Syphilis testing was recommended in 77% (69/90) of all guidance documents: 91% (30/33) in HIC documents and 68% (39/57) in LMIC documents. In LMIC guidance documents retrieved, 12 did not specify the type of testing for syphilis for people on PrEP. However, 13 recommended the combination of a treponemal and a non-treponemal test, 7 recommended non-treponemal tests only, 2 recommended a treponemal test only (rapid test) and 5 serological tests without specifying which one. Out of the 17 countries with recommendation for both treponemal and non-treponemal tests, 12 mentioned rapid diagnostic tests (RDTs) and 2 of those cited the dual HIV/syphilis test. In the case of HICs, 4 mentioned both treponemal and non-treponemal tests, 2 recommended only non-treponemal tests, 14 documents noted only the need to perform serological testing and the remaining 10 did not specify any type of test. No HICs noted RDTs.

Gonorrhoea and chlamydia testing, when mentioned, was always mentioned together. They were cited in 59% (53/90) of national guidance documents, with 88% (29/33) in HICs and 42% (24/57) in LMICs. Details on types of tests, anatomical sites of sample collection and treatment were limited, particularly in LMICs. Thirty eight per cent (9/24) of LMIC documents recommended molecular testing for gonorrhoea and chlamydia vs 66% (19/29) of HICs. Additionally, more HICs recommended sampling of extragenital sites for gonorrhoea and chlamydia testing than LMICs, 62% (18/29) and 33% (8/24), respectively.

### Frequency of STI testing

Most national guidance documents which mentioned testing for specific STIs also recommended frequency of testing. Independently of the pathogen, quarterly STI testing was the most recommended frequency in both HIC and LMIC documents, followed by periods between 3 and 6 months, and rarely on an annual basis. Sixty three per cent (19/30) of HICs recommended quarterly syphilis and 59% (17/29) gonorrhoea and chlamydia testing, while 54% of LMICs recommended quarterly testing for syphilis, gonorrhoea and chlamydia.

### STI antimicrobial resistance (AMR)

There was no mention of testing or monitoring for STI AMR in any guidance documents analysed.

### Vaccination

Vaccination against HBV infection was frequently recommended in countries’ documents, reaching 88% (29/33) in HIC and 63% (36/57) in LMIC. Conversely, vaccination against HPV was recommended by 30% (10/33) of HICs and by 14% (8/57) of LMICs.

### STI partner services

Only 16% (14/90) of all country guidance documents mentioned the need to follow-up and/or discuss partner notification within their PrEP guidance, 27% (9/33) from HICs and 9% (5/57) from LMICs. None of the 14 documents in which partner services were mentioned provided guidance on how to notify sexual partners of those diagnosed with an STI.

## Discussion

There is a unique opportunity to integrate and elevate STI services as part of PrEP programmes, particularly given the rapid increase in the number of countries recommending PrEP.^3 11 12^ Such integration would strengthen people-centred care and support a more focused and effective response to these epidemics. This study found that most countries have used this opportunity of integrating STI services within PrEP programmes by including some degree of STI testing and management in their national PrEP policy. However, particularly in South-East Asia, Eastern Mediterranean and African regions, missed opportunities for integration at the policy level were evident and could be addressed.

Testing and vaccination for HBV was recommended in most guidance documents. The frequent mention of HBV testing at baseline is recommended by WHO and does aid effective management of people taking PrEP.^11^ The high offer of HBV vaccination as part of PrEP programmes opens a programmatic door for the introduction of other vaccines for populations disproportionately affected by STIs, such as against hepatitis A virus, human papillomavirus (HPV) and mpox.^12^ Similarly, PrEP services could be an ideal platform for the targeted use of meningococcal B outer-membrane vesicle vaccines against gonorrhoea, if a recommendation is made.^12^

Testing for syphilis was recommended in most PrEP national guidance; however, rapid tests were rarely mentioned, except for some LMICs. Dual HIV/syphilis testing has been shown to be cost-effective and cost-saving in certain settings and populations.^13^ Currently, they cost less than US$1 per test and are purchased by the main international donors.^14^ As such, as reported in GAM, fast-growing number of LMICs are adopting these tests, particularly in antenatal care services. For these countries, dual HIV/syphilis testing among people on PrEP should be relatively easy to implement as systems should already be in place.^12^ Considering the increasing in national reports showing rises in syphilis incidence, particularly among men who have sex with men (MSM),^15 16^ HIV/syphilis testing could be a win-win strategy. The caveat when adopting such tests, however, is that a second non-treponemal test is required for diagnosing active syphilis, since a treponemal test alone does not differentiate priorly treated from current infection. The availability of dual treponemal and non-treponemal (T/NT) tests in LMIC is limited as reflected in their guidance documents analysed. Recognising this limitation, WHO recommends pregnant women to be tested with a rapid treponemal test and treated based on a positive test result in such context.^17^

Testing for gonorrhoea and chlamydia was not consistently recommended likely due to high prices particularly for LMICs. While high-performance, easy to use and affordable tests for chlamydia and gonorrhoea are not available in the global market, the following potential suggestions to decrease testing costs could be considered depending on context:

1. Pooling samples collected from different anatomical sites of a single individual is recommended by WHO in order not to miss extragenital infections using the same resources (ie, one single test).^18^
2. Optimising the use of available molecular platforms for tuberculosis or HIV viral load count also for gonorrhoea and chlamydia testing.^19^
3. Prioritising chlamydia testing among young women due to the potential harmful effects of an undiagnosed infection in this population.^20 21^
4. Introducing self-collection of samples, which is highly acceptable and could be used between, or in place of, clinic visits to also decrease costs and providers’ workload. Self-collection of samples for gonorrhoea and chlamydia is also recommended by WHO.^22^
5. Reducing the frequency of some STI testing from quarterly to every 6 months.^23^

In our review, most countries recommended testing for STIs at baseline and quarterly follow-ups. Regular asymptomatic screening has the benefit of early detection and treatment, and potential reduction of further transmission, especially for syphilis. However, unlike syphilis, gonorrhoea and chlamydia infections can be cleared by the immune system and, consequently, treating all asymptomatic infections implies treating several infections that would self-resolve without treatment, resulting in risk for AMR without additional individual benefits, particularly for MSM.^24 25^ Therefore, reducing gonorrhoea and chlamydia screening frequency is likely to reduce the number of asymptomatic infections that are unnecessarily treated.

Furthermore, a recent systematic review suggested that testing people on PrEP every 6 months for gonorrhoea and chlamydia seems to be more feasible and less costly yielding similar outcomes.^25^ From a public health perspective, the impact of frequent screening for these two pathogens on reducing population level prevalence is not always clear in all contexts and, from a person-centred approach, individuals may experience stigma and adverse psychological effects related to unnecessary STI diagnosis and treatment.^26–28^ For MSM in HIC, it is also important to note recent discussions on the balance of benefits versus harm in regular asymptomatic testing and treatment for chlamydia.^26 27^ However, regular testing for chlamydia among young women remains the standard of care recommended by several countries.^20^ More data are needed to best define the frequency of testing for different populations and contexts.^23^

The growing gonococcal AMR is of high global concern requiring immediate attention. Improved prevention and early diagnosis among those at higher risk of infection are crucial interventions to address AMR.^29^ PrEP services may provide a valuable opportunity to support national AMR surveillance as this target population is already linked to health services.^12^ Further work is needed to facilitate this integration.

Partner services for STIs, also known as partner notification or contact tracing, are a main pillar of STI management and response. Partner notification was rarely mentioned in the reviewed guidance but this could partially be explained as guidance for partner notification related to STIs may be included in national STI management guidelines rather than in PrEP guidelines.

Since 2016, WHO has recommended a range of evidence-based partner services strategies to support those diagnosed with HIV to notify their sexual partners^30^ and more recently to partners of those diagnosed with bacterial STIs (upcoming WHO publication). In facilities where HIV partner services strategies are already in place, the inclusion of other STIs would require minimal additional resources when compared with establishing such services independently of HIV. For some curable STIs, expedited partner therapy has been shown to be effective and acceptable depending on context and should be also considered.^31^

There are several limitations in our study. First, this review included only guidance documents available to the reviewers, thus there might be some countries that have guidance but were excluded despite efforts to obtain documents. Second, when PrEP guidance documents did not include information on STIs but referred providers to national STI management guidelines, these were not reviewed or included in the analysis, as we started from the assumption that this is a missed opportunity not to recommend STI services when offering PrEP. Third, our review was limited to national guidance documents, thus subnational level documents were not included. Finally, information may have been lost when documents were translated into English.

## Conclusion

To the best of our knowledge, this is the first review of national PrEP policies that focused on the integration of STI services recommendations for people seeking or taking PrEP. The results of this review highlight the gaps and missed opportunities in these national normative guidance documents to support addressing, prioritising, focusing and integrating evidence-based recommendations related to STI control and management for those at higher risk of infection. It also provides baseline data to monitor progress towards integrating STI interventions in national PrEP guidelines.

Countries implementing PrEP, but with limited resources to add recommendations on STI interventions, should consider using a stepwise approach for STI service integration, such as initiating by prompting clients for STI signs and symptoms and by adopting dual HIV/syphilis rapid testing.^12^ Integration of services should always be informed by the local context, including epidemiology, availability of resources and acceptability by users and providers.

Integrating STI services for people who seek or use PrEP offers an opportunity to improve person-centred care and address STI epidemics alongside HIV by fostering synergies and efficiencies in the provision of HIV services and contributing towards the 2030 goals of Global health sector strategies on HIV, viral hepatitis and STIs, 2022–2030.^2^

## Data Availability

Data is available upon reasonable request. Data set used to generate tables and results will be made publically available in the future.
