# Supplementary material for: Missed opportunities for guidance on sexually transmitted infection services: a global review of national HIV PrEP guidance
Source: Sex Transm Infect. 2024 Jun 19;100(6):343–8. doi: 10.1136/sextrans-2023-056081 (PMC11347216; doi:10.1136/sextrans-2023-056081)
Supplement: Supplementary data [file sextrans-2023-056081supp001.pdf]

Supplement 1.

TABLE S1: List of variables used for data extraction on STIs recommended for people on PrEP. Only those used in the analysis presented are listed.

**Information on guidance documents**
WHO Region
Name of Member State
Country income level according to the World Bank classification[i]
National HIV guidance (Yes/No)
Name of the HIV guidance document and weblink
Date of most recent national HIV guidance document
HIV guidance document includes information on PrEP (Yes/No)
Stand-alone PrEP guidance available (Yes/No)
Name of the most recent national PrEP guidance document and weblink
Date of most recent PrEP guidance document
Types of population eligible for PrEP*: Key populations (i.e., MSM, sex workers, transgender people, people who use drugs and incarcerated individuals), HIV serodiscordant couples, adolescents and young people (boys and girls), none mentioned.
Place of STI information:<br>- Yes, included in HIV national guidance<br>– Yes, stand-alone PrEP guidance<br>– Yes, mention STI but provides no STI information<br>– Refers reader to other STI guidance in the context of PrEP<br>- No information on STIs found
**Recommended interventions**
Partner services: any strategy mentioned to reach sexual partners of PrEP users diagnosed with a STI or who presented with signs or symptoms (Yes/No)
Types of STI case management: syndromic only, syndromic + syphilis testing, etiologic only, both (etiologic plus syndromic)
Syphilis testing at baseline (Yes/No)
Syphilis testing frequency
Types of syphilis test: treponemal, non-treponemal, both, order of tests, rapid tests and types of rapid tests
Syphilis treatment (Yes/No)
Gonorrhoea testing at baseline (Yes/No)
Anatomical site of sample collection: oropharyngeal, anorectum, urethral, vulvovaginal
Gonorrhoea testing frequency
Types of gonorrhoea test (molecular, culture, microscopy, others)
Treatment for gonorrhea (Yes/No)
Antimicrobial resistance (Yes/No)
Chlamydia testing at baseline (Yes/No)

Spielman E, *et al. Sex Transm Infect* 2024; 100:343–348. doi: 10.1136/sextrans-2023-056081

Anatomical site of sample collection: oropharyngeal, anorectum, urethral, vulvovaginal
Chlamydia testing frequency
Types of chlamydia test (molecular, rapid tests, others)
Treatment for chlamydia (Yes/No)
Hepatis B baseline testing (HbsAg**) (Yes/No)
Hepatitis B vaccination and circumstance (e.g., HbsAg* negative, prior vaccination, any)
Human papilloma virus (HPV) vaccination (Yes/No)
HPV molecular testing (Yes/No)
Screening for pre-cancerous cells** (Yes/No)
Other STIs* (Yes/No)
Notes to support interpretation of data analysis

Notes: *Hepatitis B surface antigen.: ** Results not presented;

[i] Income level according to the World Bank: # http://databank.worldbank.org/data/download/site-content/CLASS.xlsx

Spielman E, *et al. Sex Transm Infect* 2024; 100:343–348. doi: 10.1136/sextrans-2023-056081
